# Conductometric Soot Sensors: Internally Caused Thermophoresis as an Important Undesired Side Effect

**DOI:** 10.3390/s18103531

**Published:** 2018-10-19

**Authors:** Gunter Hagen, Christoph Spannbauer, Markus Feulner, Jaroslaw Kita, Andreas Müller, Ralf Moos

**Affiliations:** Bayreuth Engine Research Center (BERC), Department of Functional Materials, University of Bayreuth, 95440 Bayreuth, Germany

**Keywords:** particulate matter sensors, exhaust gas aftertreatment, particulate filter, soot deposition, electrophoresis, thermophoresis, dynamometer

## Abstract

Particulate matter sensors are of interest for application in the exhaust of any combustion processes, especially for automotive aftertreatment systems. Conductometric soot sensors have been serialized recently. They comprise planar interdigital electrodes (IDE) on an insulating substrate. Between the IDEs, a voltage is applied. Soot deposition is accelerated by the resulting electric field due to electrophoresis. With increasing soot deposition, the conductance between the IDE increases. The timely derivative of the conductance can serve as a sensor signal, being a function of the deposition rate. An increasing voltage between the IDE would be useful for detecting low particle exhausts. In the present study, the influence of the applied voltage and the sensor temperature on the soot deposition is investigated. It turned out that the maximum voltage is limited, since the soot film is heated by the resulting current. An internally caused thermophoresis that reduces the rate of soot deposition on the substrate follows. It reduces both the linearity of the response and the sensitivity. These findings may be helpful for the further development of conductometric soot sensors for automotive exhausts, probably also to determine real driving emissions of particulate matter.

## 1. Introduction

Fine dust and particulate matter (PM) emissions from combustion processes, especially from diesel combustion, may cause serious health problems. The exhaust gas aftertreatment of diesel engines becomes increasingly complicated due to tightening emission standards [1,2,3]. The determination of vehicle emissions during defined driving cycles on chassis dynamometers is going to be replaced by real-driving emissions measurements [4]. Therefore, prospective needs will concern reliable on-board measurement systems—also for PM [5,6].

Particle abatement with diesel particulate filters (DPF) is indispensable in future [5,6]. Additionally, in the field of gasoline fueled passenger cars, the PM issue may become even more serious, since nano-sized soot emissions have to be avoided [7]. In the past, particle mass was the key target, but recently, a particulate number limit has also been introduced. Therefore, gasoline particulate filters (GPF) have also been serialized [8,9]. In addition to that, on-board-diagnostics (OBD) has to ensure permanently the correct functionality of all exhaust gas aftertreatment systems, which is here the filtering efficiency of particulate filters [10,11,12,13,14].

For PM aftertreatment, as the current state-of-the-art, ceramic wall-flow filters are applied in the exhaust pipe. Soot from the raw exhaust is trapped over a certain time span, but the filter has to be regenerated during operation to avoid clogging. Therefore, soot load prediction models based on differential pressure sensors signals and engine characteristic maps are in serial use to estimate the filter loading state [12]. The more exactly the filter loading is known, the more efficiently the system can be operated. Filter regeneration at higher exhaust gas temperatures to burn off the trapped soot causes higher fuel consumption and should be carried out only when necessary [15,16]. A promising novel approach uses microwave sensors to determine directly the filter loading degree in operation [12,17,18,19].

**Figure 1:**
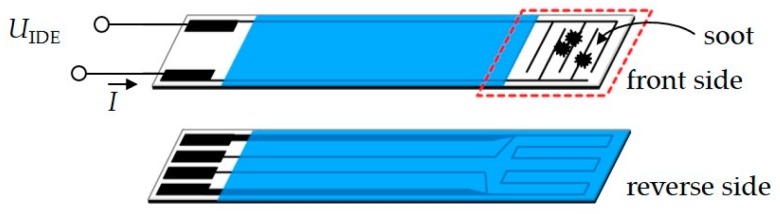
Sketch of the sensor setup: The sensors front side comprises interdigital electrodes (IDE) where soot deposits yield electrical conductive pathways over the spacing. On the reverse side, a four-wire thick-film heater allows temperature-controlled sensor regeneration as well as temperature measurement and temperature adjustment during soot deposition.

For OBD-purposes, soot sensors are useful tools. Several principles are in the research or development state [20,21,22,23,24,25,26]. Conductometric (sometimes also called “resistive”) soot sensors are very often discussed, in industry [20,27,28,29] and academia [30,31,32,33]. A typical sensor is sketched in Figure 1. This device is explained in detail, since it is also used for the experiments described below.

On the upper side of an insulating, high temperature stable alumina substrate, interdigital electrodes (IDE) are screen-printed with Pt paste (LPA88-11S, Heraeus, Hanau, Germany), dried and fired. The electrode area is about 5 mm × 5 mm and comprises lines and spaces, both of 100 µm width. On the reverse side of the substrate, a platinum thick-film structure is integrated. It is designed in a way that the temperature distribution on the sensor side is homogenous, if one uses the platinum thick-film structure as a heater [34]. Since it is designed in a special four-wire technique, the tip resistance can be measured and the temperature can be deduced from the tip resistance. The (almost linear) correlation function between heater resistance and sensor temperature is calibrated for each sensor in the lab before using the device. The heater structure provides two different functionalities. Firstly, it can be used for heating, i.e., for generating a homogenous temperature distribution in the IDE area either to regenerate the sensor surface by oxidation of soot at high temperatures (600 °C) or to heat the sensor tip to a desired temperature. Secondly, it enables measuring the sensor temperatures during soot deposition, which is, in fact—if only a very small heater current is applied—also a measurement of the actual exhaust gas temperature. All feed lines as well as the heater structure are covered by a glass ceramic passivation (QM 42, DuPont, CCI Eurolam, Dreieich, Germany) to avoid interconnections by soot particles. More details on the sensor setup can be found in previous publications [35,36].

**Figure 2:**
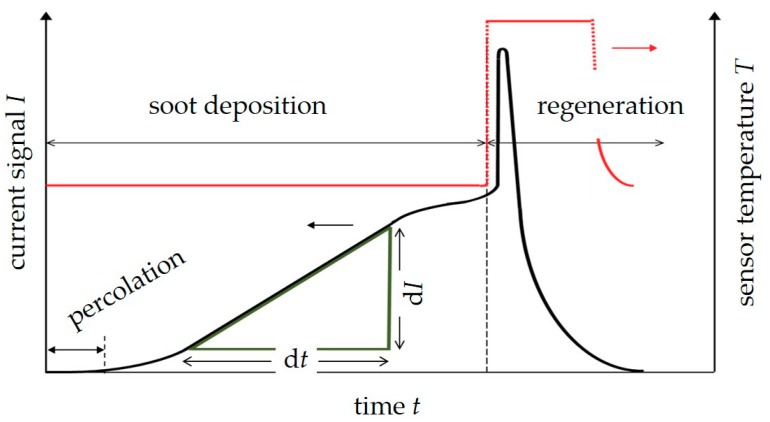
Typical shape of the sensor’s raw signal during one sensing cycle with increasing current *I* after a certain “blind” time before the percolation threshold is reached and a regeneration peak after increasing the sensor’s temperature for soot burn off. The regeneration peak is due to the increased soot conductivity when heating up. The slope of the linear increasing current during soot deposition, which is proportional to the conductance, often acts as the sensor signal.

Several modifications, especially for serial applications, have been reported in literature [20,26,27,28,29,31,32]. Not depending on the manufacturer or research lab, most of the conductometric soot sensors are operated similarly. By applying a voltage *U*_IDE_ to the IDEs, an electrical current *I* can be measured as soon as the deposited soot particles form a first electrical conductive percolation path between these electrodes. After this blind time, with proceeding soot deposition, the current increases. In other words, the sensor is an integrating device following the dosimeter principle with an instantaneous electrical readout, as described in [37]. During a subsequent regeneration, the sensor is heated to several hundred °C to burn off the soot. When cooled down to ambient temperature, i.e., to exhaust temperature, a new measurement cycle can start, again beginning with a blind time. In addition to a conductometric readout, capacitive measurements have also been investigated [23,24]. A typical operation mode of a conductometric soot sensor is described in Figure 2.

In our former work, we already showed the possibility for soot mass flow detection with these dosimeter-like conductometric sensors. A direct relation between the soot mass flow in the raw exhaust of a diesel engine and the timely derivative of the current (d*I*/d*t*) between the IDE when a constant voltage is applied was found [35,36].

In several contributions, the influence of the applied voltage was already found to be a significant parameter for soot deposition. Higher electrical fields interact with the charged soot particles. Another option is that electrical charges might be induced by the electrical field. Anyway, the soot is accelerated to the sensor’s surface by the Coulomb forces and thus soot collection is enhanced electrophoretically [38,39,40,41,42,43]. However, in order to reliably determine particulate mass or particle number, a deeper understanding of the deposition mechanisms on the sensor surface is needed.

Soot is a highly complex matter. Characteristics like morphology, carbon content, adhered volatile organic components content or water, or its electrical charge will also influence the interaction with a measuring device [7,44,45,46,47,48,49]. For a further knowledge-based development of conductometric soot sensors, the influences of the applied voltage and of the sensor temperature on the soot deposition needs to be investigated. In order to keep flexibility with changing geometries, electrode designs, sensor orientations, housings, or to even be able to manufacture planar capacitive devices [24], we used our own sensor structure as shown in Figure 1. It is fully manufactured in thick-film technology, which is very close to typical production type sensors [27,28]. It is expected that our findings can be transferred to commercial sensors as well.

## 2. Sensor Design and Experimental Setup

It is the intention of the present study to contribute to a better understanding of factors that influence soot deposition on such sensors. Therefore, simple conductometric sensor devices were built as shown in Figure 1 and operated as explained above. In addition to that, in some experiments, the platinum film structure is used to adjust a defined temperature on the sensor tip, i.e., on the IDE, during soot deposition. Here, the sensor temperature is set to a few degrees higher than the exhaust gas temperature to study thermophoresis effects. The results are reported in the second part of this study.

After wiring and housing, the sensors were mounted into the exhaust pipe. Here, the sensors either face the gas flow with its IDE area or are oriented in a way that the sensor is mounted exactly perpendicular to the exhaust flow with the electrode area being behind the substrate in the shadow zone. No protection caps were used for these experiments. The sensor current and other parameters like heater resistance, applied voltage between the IDE (*U*_IDE_), or heater voltage and heater current during active heating were recorded with a digital multimeter (2700 series, Keithley, Tektronix, Munich, Germany).

**Figure 3:**
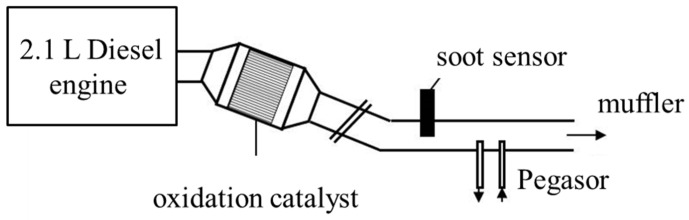
Setup for real exhaust measurements with a 2.1 L diesel engine.

Experiments in the dynamometer test bench, i.e., in real exhausts, were conducted in the exhaust pipe of a 2.1 L diesel engine (Figure 3). To change the amount of soot during operation, the boost pressure *p*_boost_ was varied between 1.25 bar and 1.15 bar. All other engine parameters were kept constant (1000 rpm, 25% accelerator pedal position, injection pressure *p*_inj_ = 550 bar). Most of the tests were conducted several times at different days to get an idea of the repeatability of the soot generation and to distinguish scattering (coming from soot concentration variations) from signal changes (resulting from the influencing parameters to be investigated). Particle concentration data were obtained by a commercial soot nanoparticle measurement device (Pegasor) simultaneously during all experiments.

To investigate the effect of soot deposition, the sensors were operated under constant exhaust conditions during the soot-collecting phase. A certain dc voltage was applied between the electrodes (*U*_IDE_) and the resulting current *I* was recorded. After that, soot regeneration was initiated by heating up the device. Regeneration leads to the well-known peak-shape in the current signal as shown in Figure 2 (and also for instance in [12,27,36]) followed by a zero current phase, when all soot is burned off. A new soot measurement cycle is then started. Within each engine operation point, at least three cycles were recorded unless otherwise stated in the respective section.

As a first step, dynamometer test at different engine operation points by varying the boost pressure to achieve different soot concentrations were conducted. One series included three sensor loading cycles, at three different soot concentrations, respectively. For each single measurement cycle, the sensor signal d*I*/d*t* was calculated as a slope value in mA/s (only the linear part of the *I*(*t*)-curve was taken to calculate the slope, as indicated in Figure 2). So, one measurement series provided nine data points, three for each soot concentration. These series were repeated several times at different days, with the dc voltage applied between the sensor electrodes being varied from 20 to 60 V.

## 3. Results and Discussion for Real Exhaust Measurements

### 3.1. Influence of the Applied Voltage/Electrophoresis

**Figure 4:**
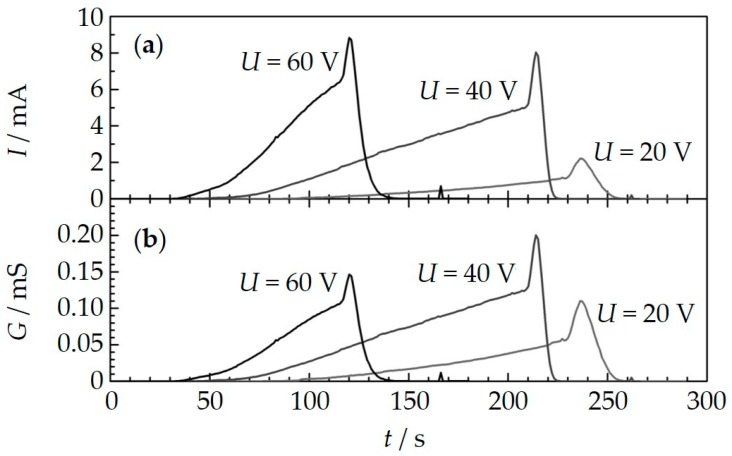
Raw data *I*(*t*) ((**a**) and calculated conductance data *G* (**b**) for three measurement cycles (soot collection with different voltages and subsequent regeneration) under similar conditions (soot concentration = 17 mg/m^3^). The curves are shifted to a common starting time that is the end of the prior regeneration phase.

In Figure 4, three typical and representative single sensor loading cycles under almost equal soot concentrations (17 mg/m^3^) are shown. The voltage between the IDEs *U*_IDE_ was varied. The data are shifted to a joint starting time *t* = 0, which is the end of the previous regeneration procedure. The raw data *I*(*t*) already show the described effect: a higher voltage leads to a higher slope of the current. At first glance, one would attribute this to the higher voltage that drives a higher current in agreement with Ohm’s law. However, the (calculated) conductance curves (*G* = *I*/*U*_IDE_) still show the same behavior: the higher the voltage, the higher the slope of the conductance d*G*/d*t*. In other words, the soot deposition rate is in fact influenced by the applied voltage and one may assume that the amount of deposited soot on the sensor surface increases with the applied voltage. The data shown here are taken with a sensor oriented with electrodes facing the gas flow, but the findings are very similar for the opposite mounting.

The percolation time (or blind time) also varies with the applied voltage and supports the previous findings. It can be determined directly from the current and/or the conductance curves. For lower applied voltages, the blind time until a first current appears increases. It takes nearly 100 s for 20 V and just 40 s for 60 V until the first current flows. Hence, higher voltages support the formation of conducting paths between the electrodes.

The effect of electrical field-supported soot deposition is well known. Soot particles from exhausts are electrically charged and are be attracted to the electrodes by a potential difference in such setups [38,39,40,41,42,43]. Detailed information on the charging of soot particles and electrophoresis are given in [50].

**Figure 5:**
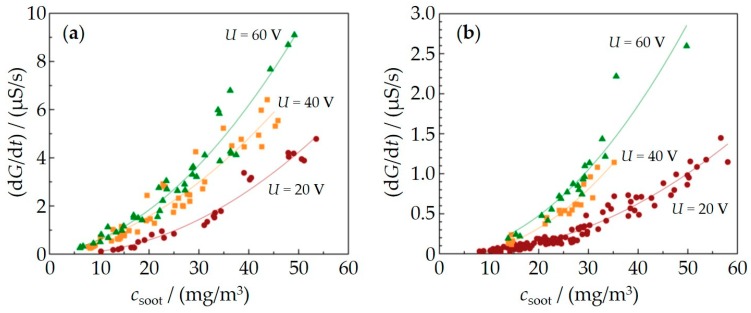
Sensor calibration curves (calculated conductance d*G*/d*t* vs. soot concentration) for different types of sensor orientation in the exhaust, either IDE facing the gas flow (**a**) or in the opposite mounting position (**b**).

Figure 5 shows the sensor calibration curves, which include the timely derivatives of the conductance d*G*/d*t* of a measurement cycle for each point plotted over the soot concentration measured by the Pegasor sensing device (values given in mg/m^3^). Monotonous correlations between d*G*/d*t* and soot concentrations are found for both types of sensor orientation. Furthermore, the provided conductance data are normalized to a common temperature of 300 °C. Higher voltages result in a higher sensor signal. All these findings agree with the literature [35].

The normalization was carried out by a linear calibration curve of the temperature dependency of the conductance. For that purpose, a sensor was operated in the exhaust and soot was deposited for 150 s at *U* = 60 V. This produced a typical soot layer on the electrodes. The sensor was then taken out of the exhaust. Using the internal heater, temperatures between 150 °C and 375 °C were adjusted and the conductances were measured for 20, 40, and 60 V, respectively. In all experiments, a temperature dependency of d*G*/d*T* of ca. 0.3 µS/°C was found.

To obtain higher sensor signals and a reduced percolation time, electrophoresis has to be increased by a higher voltage. This causes an interesting phenomenon that is described in the following section.

### 3.2. Influence of Thermophoresis

**Figure 6:**
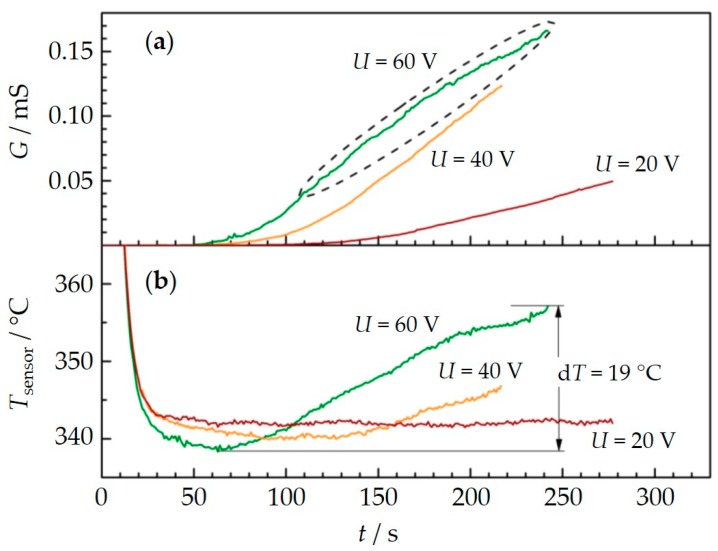
Conductance over time during soot deposition for three cycles with different voltages *U*_IDE_ between the sensing electrodes (**a**) and corresponding sensor temperature determined by the reverse side platinum meander element (**b**). The engine operation point was kept constant during the experiment (soot concentration = 30 mg/m^3^).

In Figure 6a (upper graph), raw data of the conductance for three measurement cycles with subsequent regeneration for applied voltages between the interdigital electrodes of 60, 40, 20 V are shown. The engine operation point and, therefore, both the exhaust temperature and the soot concentration (30 mg/m^3^) were kept constant during the experiment. According to thermocouple data, the exhaust temperature varied only in a range of ±5 K. The increasing part of the conductance is of special interest here (area highlighted by a dashed ellipse). As already known from Figure 3, the slope of the conductance curve increases with increasing voltages and first percolation paths occur faster. Astonishingly, for 60 V, the slope decreases, at least in the second half of the soot deposition period, whereas for 40 V and for 20 V the slope remains constant.

The respective temperature of the sensor element, which was measured by the internal heater structure during the whole experiment, is plotted in Figure 6b. In general, this temperature represents the exhaust temperature. Considering the temperature at 50 s, i.e., after sufficient cooling from regeneration temperature, one finds that the operation conditions are relatively constant and the exhaust temperature varies only in the range of 5 °C. The eye-catching result in this diagram is the increase of the sensor temperature during soot deposition for 60 V of about 19 °C. When 40 V are applied for soot deposition, the sensor temperature increases a little less but still by 9 °C. No temperature increase can be seen for *U*_IDE_ = 20 V. This suggests that a temperature increase of the sensor is responsible for the decreasing slope of the conductance in Figure 6a.

**Figure 7:**
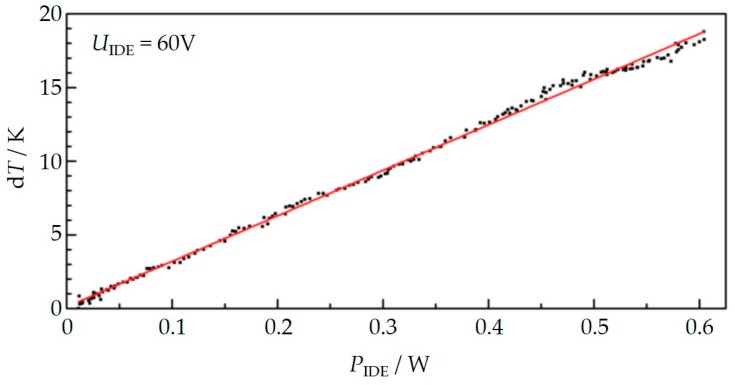
Internal self-heating of the device: Correlation of the increase of the sensor temperature (determined from the resistance of the platinum structure) and the calculated electrical power on the sensing electrodes during soot deposition when 60 V were applied, calculated from the data in Figure 6.

Since a temperature increase of the exhaust gas can be ruled out, an internal heating effect must be responsible for the observed behavior. If one calculates the power *P*_IDE_ (*P* = *U*_IDE_·*I* = *U*_IDE_^2^·*G)* that generates Joule’s heat between the sensing electrodes and plots it vs the observed temperature increase, one obtains Figure 7 (for *U*_IDE_ = 60 V). In other words, during soot deposition, the power *P*, which generates heat directly in the deposited soot, increases. At the beginning, the conductance *G* is zero since no current flows. Since there is no additional power *P*, there is no temperature increase. The higher the soot loading between the IDE, the higher is the conductance and the more heat is generated. At the end after 250 s, a temperature increase of 19 K occurs. According to Figure 7, applying 60 V between the electrodes leads then to a heat generation of 0.6 W. If we compare that with our experiences concerning the heater structure, materials data and simulations on similar sensor setups [51], an increase of 19 K seems plausible. As a conclusion, the temperature increase with higher applied voltages has a direct and an indirect origin. Due to the higher voltage, the soot deposition rate is higher due to the electrophoretic effect. This causes thicker soot layers that lead to a higher conductance of the soot films. In addition, the generated heat increases quadratically with the voltage.

Therefore, we conclude that the internally generated heat reduces the rate of particulate matter deposition on the sensor surface due to thermophoresis [26,52,53], in other words, internally generated thermophoresis counteracts electrophoresis. This would explain the decreasing slope in the conductance curve as it has been found out in Figure 5a for 60 V.

**Figure 8:**
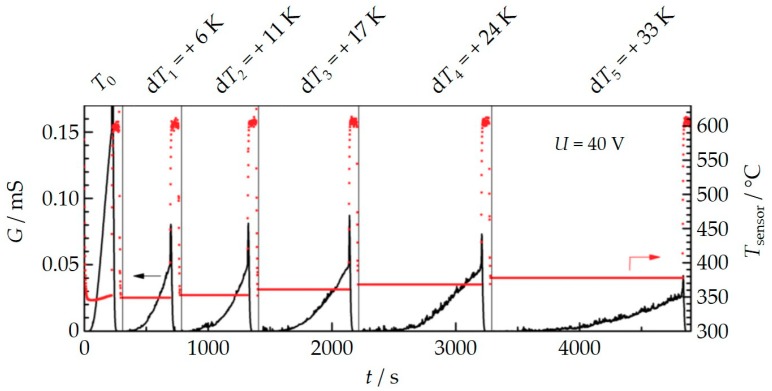
Conductance *G* for several soot deposition cycles at *U*_IDE_ = 40 V during constant engine operation. After regeneration, an additional small power was applied to the sensor to establish a slightly higher temperature of the sensor tip compared to the exhaust temperature. This temperature difference d*T* = *T*_sensor_ − *T*_exhaust_ that was kept constant during each soot deposition phase.

Another experiment was conducted to investigate this “internally caused” thermophoresis effect further. Again, a sensor was constantly operated in the indirect mode with the heater structure facing the gas flow, now with 40 V applied between the electrodes. The engine was operated under constant speed and load. We conducted several soot deposition cycles, one after the other. After each regeneration procedure, the heater was not completely switched off, but set to a certain temperature. As a result, the sensor temperature (*T*_sensor_) during the soot deposition phase could be deliberately increased by small values, d*T*, compared to the exhaust temperature (*T*_exhaust_). The sensor temperature was calculated from the four-wire resistance of the internal platinum structure. In the first cycle, no additional heating was applied (d*T* = *T*_sensor_ − *T*_exhaust_ = 0 K). In the following cycles, the temperature was increased stepwise to d*T* = 6, 11, 17, 24, and 33 K. The results are shown in Figure 8.

It turned out that the higher the sensor temperature is, the longer it takes until the first percolation sets in and the lower the slope of the conductance curve is during soot deposition.

**Figure 9:**
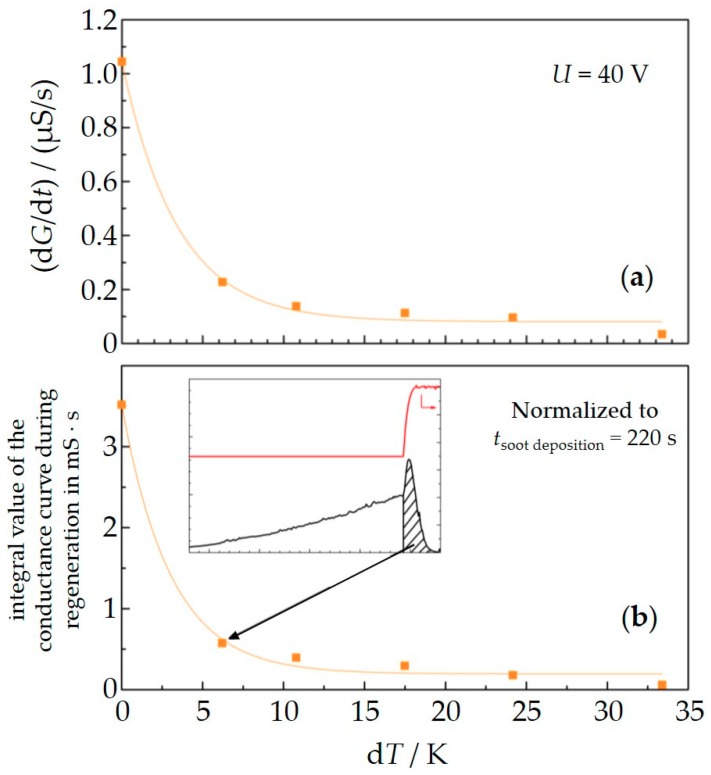
Slope of the conductance d*G*/d*t* (evaluated from the data in Figure 8) vs. temperature difference between sensor and exhaust gas d*T* (**a**). The normalized integral values of the conductance curve during regeneration show similar behavior (**b**). The inset shows the calculation for the integral value exemplarily.

In Figure 9a, the slopes of the conductance d*G*/d*t*, as they were derived from Figure 8, are plotted over the temperature difference d*T*. Since d*G*/d*t* is a measure for the amount of soot that gets deposited on the sensor per time interval, the monotonously decreasing curve supports the assumption that “internally caused” thermophoresis is an effect that has to be considered when deducing soot concentrations from the slope of the conductance d*G*/d*t*.

In addition to that, this assumption was verified by another analysis. During regeneration, the current and correspondingly the conductance shows a “peak” behavior. The step change in sensor temperature up to 600 °C leads to an almost sudden conductance increase due to the enhanced soot conductivity, followed by an abrupt decline when the soot gets oxidized and percolation is interrupted. The conductance reaches zero as soon as the soot is burned off. This behavior is well known and often described in literature [12,27,36]. The area below the peak of the conductance curve during regeneration may be correlated to the amount of soot that has been deposited right before the regeneration starts as both effects, the increasing conductance as well as the declining curve, depend on the thickness of the soot layer or its mass, respectively. In case of less deposited soot, the increasing part of the peak during heating up is smaller (less influence of temperature dependent conductivity increase). Furthermore, the declining part of the peak starts faster (less amount will be burned off earlier). Therefore, the peak area is small. Analogously, a higher amount of soot causes a higher current increase, the total oxidation takes longer and as a result, the peak area is larger. So, it should be possible to estimate the final soot loading (i.e., the amount of deposited soot mass) on the sensor surface before regeneration by evaluation of the peak area. We now plot these integral values of the conductance curve during regeneration vs. the temperature difference during soot deposition. Since not only the parameters *T*_sensor_ or the applied voltage *U*_IDE_ determine the amount of soot on the sensor surface, but also the individual time intervals for soot deposition for each single measurement, we have to normalize the integral value results to a similar time span of soot deposition to compare different measurement/regeneration cycles. This is done by the linear relation of conductance over time for each measurement cycle. All values refer now to a soot loading interval of 220 s. Figure 9b shows the correlation of the normalized integral values vs. d*T*. Here also, the exponential decrease occurs with very similar parameters as found before (exp(−0.3x)). Therefore, the evaluation of the peak area during regeneration can be used as an additional indicator of the amount of deposited soot right before regeneration. This method helps to understand thermophoresis effects when using synthetic soot (e.g., from CAST devices). Here, the conductivity of the soot does not linearly increase with temperature. Therefore, the conductance data are not sufficient to describe the amount of deposited soot [54].

In conclusion, thermophoresis strongly affects the rate of soot deposition on the sensor device. Whereas a higher voltage applied between the electrodes enhances soot deposition due to electrophoresis, a higher sensor temperature (which might be also caused by the applied high voltages between the electrodes) favors thermophoresis.

This effect might also be practically used. It should be possible to “adjust” the measurement ranges of soot sensors. Very fine electrode structures, for example, which allow detection of ultra-low soot concentrations (easy and fast path building) might be operated at higher temperatures in case of atmospheres with high amounts of soot.

## 4. Conclusions and Outlook

The present contribution considered the effect of different voltages that generate different electrical fields to support the attraction of soot particles to enhance the deposition on the surface of planar conductometric soot sensors. In contrast to other studies, we carefully investigated the effect of internal heating of the soot due to higher applied voltages. The internally caused thermophoresis is an effect that hinders the application of higher voltages between the electrodes in conductometric soot sensors. Both mechanisms occur in real exhausts as well as in synthetic conditions; the results coincide in general. However, using synthetic soot causes some challenges which should be discussed in a separate paper.

Advantageously, these parameters might be used to influence directly and knowledge-based the soot deposition or interaction with a sensor device. This could help to adjust even the sensitivity of soot sensors and its use in different atmospheres with varying soot concentrations.

As a future goal, we intend to develop soot sensors that are sensitive to certain soot fractions. Therefore, ongoing work will focus on lab measurements with defined particle fractions. Variations will be necessary also concerning the IDE geometry or its layout, the mounting position, or even the sensor principle. The possibilities of influencing soot adsorption by the device’s operation strategy (voltage and temperature) might be adaptable to “select” defined soot fractions, e.g., to discriminate between different soot fractions. Here, the exhaust gas temperature and the exhaust velocity should be taken into account. Recently published investigations on modeling particle deposition mechanisms [55] indicate an influence of these parameters on the question of whether electrophoresis or thermophoresis plays the major role.
